# Multiscale Models of the Antimicrobial Peptide Protegrin-1 on Gram-Negative Bacteria Membranes

**DOI:** 10.3390/ijms130911000

**Published:** 2012-09-05

**Authors:** Dan S. Bolintineanu, Victor Vivcharuk, Yiannis N. Kaznessis

**Affiliations:** Department of Chemical Engineering and Materials Science, University of Minnesota, 421 Washington Ave SE, Minneapolis, MN 55455, USA; E-Mails: dan.bolintineanu@gmail.com (D.S.B.); vivch001@gmail.com (V.V.)

**Keywords:** antimicrobial peptides, peptide-membrane interactions, ion transport, protegrin, multiscale models, molecular simulations

## Abstract

Antimicrobial peptides (AMPs) are naturally-occurring molecules that exhibit strong antibiotic properties against numerous infectious bacterial strains. Because of their unique mechanism of action, they have been touted as a potential source for novel antibiotic drugs. We present a summary of computational investigations in our lab aimed at understanding this unique mechanism of action, in particular the development of models that provide a quantitative connection between molecular-level biophysical phenomena and relevant biological effects. Our work is focused on protegrins, a potent class of AMPs that attack bacteria by associating with the bacterial membrane and forming transmembrane pores that facilitate the unrestricted transport of ions. Using fully atomistic molecular dynamics simulations, we have computed the thermodynamics of peptide-membrane association and insertion, as well as peptide aggregation. We also present a multi-scale analysis of the ion transport properties of protegrin pores, ranging from atomistic molecular dynamics simulations to mesoscale continuum models of single-pore electrodiffusion to models of transient ion transport from bacterial cells. Overall, this work provides a quantitative mechanistic description of the mechanism of action of protegrin antimicrobial peptides across multiple length and time scales.

## 1. Introduction

The prevalence of antibiotic-resistant bacteria has emerged as a major global public health crisis [1]. Despite the growing number of bacterial strains that have developed resistance to conventional antibiotic treatments, relatively few novel therapeutic compounds have been introduced in recent years. A promising solution to this crisis comes from antimicrobial peptides (AMPs), which are small, naturally-occurring proteins with unusually strong antibiotic properties [2]. AMPs act as a first line of defense against invading pathogens as part of the innate immune systems of countless organisms, including humans. Although they exhibit an immense variety of structural and physicochemical features, AMPs are typically composed of 10–50 amino acids, possess a net positive charge at physiological pH, and exhibit some degree of amphipathicity. The interested reader is referred to [2,3] for excellent reviews of AMP research across multiple structural classes.

**Figure 1:**
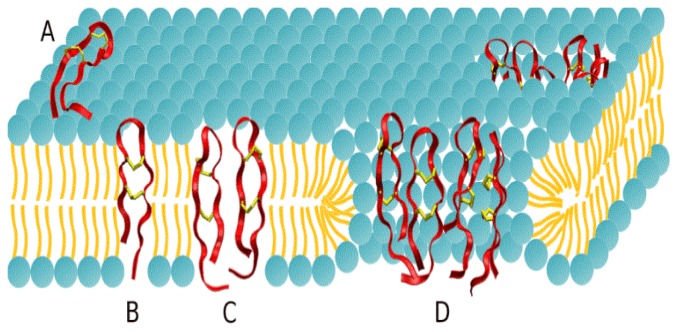
The proposed mechanism of action of Protegrin 1. Peptides (depicted in red ribbons, with the two disulfide bonds shown as yellow sticks) adsorb to the membrane surface (**A**), insert into the hydrophobic core in a transmembrane orientation (**B**), and form dimers (**C**). Peptide dimers oligomerize, likely as four or five pairs, to form water-filled, transmembrane pores (**D**) that lead to unrestricted ion transport and cell death. From [11] with permission.

In order to provide a detailed analysis of the AMP mechanism of action, we have focused our efforts on protegrin, a particularly potent peptide initially isolated in pigs [4]. The balance of the evidence suggests that protegrin and many other AMPs act primarily by interacting with and disrupting the plasma membrane of bacteria [5–11]. As a result, a great deal of effort has been expended to investigate the interactions of AMPs with cell membranes as well as model lipid bilayer membranes. Protegrin 1 (PG-1) is an excellent model peptide because it has been the subject of numerous such studies, as well as having a promising therapeutic profile. The key steps in the bactericidal mechanism of action of PG-1 are summarized in Figure 1.

In a recent review [11], we have summarized the growing body of literature dealing with computational investigations of protegrins. In the present manuscript, we recapitulate some recent work from our group in this area, with a focus on the development and implementation of multiscale modeling strategies.

## 2. Results and Discussion

### 2.1. Potential of Mean Force Calculations

**Figure 2:**
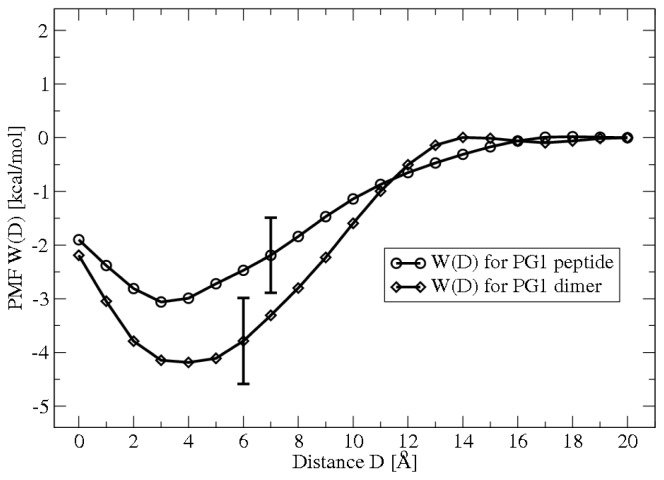
PMFs of adsorption for protegrin monomers and dimers on 1:3 POPE:POPG lipid bilayers. D is the separation distance between the PG-1 center of mass and the phosphate plane of the upper leaflet of the membrane. From [12] with permission.

The study of protein-membrane systems by experimental techniques is cumbersome due to the extremely fast time scales and small length scales of the relevant physical phenomena. To address these shortcomings, we have carried out molecular dynamics (MD) simulations coupled to free energy simulation techniques to compute various potentials of mean force (PMFs) relevant to protegrin membrane association. These simulations consist of fully atomistic representations of solvated peptide-membrane systems, in which different reaction coordinates are constrained. In the case of peptide adsorption, the relevant reaction coordinate is simply the distance between the peptide and the membrane; the resulting PMF represents the free energy of the system as a function of this distance. The details of these methods are largely omitted here, and the interested reader is referred to [12,13]. Figure 2 below shows the PMF for monomer and dimer adsorption; PMFs for dimerization in different environments are shown in [13].

These and similar results provide the most detailed quantitative measure of these distinct membrane-association processes available to date. Clearly, we can infer that monomer and dimer adsorption are both favorable, with free energies of adsorption of approximately −2.5 kcal/mol and −4.5 kcal/mol, respectively. Further analysis of the PMFs showed that adsorption is driven largely by the entropy gain associated with counterion release from the membrane [12]. Similarly, dimerization is favorable, and shows no kinetic barriers [13]. Very recently, we have also computed the PMF of peptide insertion. In this case, a barrier of +6 kcal/mol is observed, and the insertion is highly favorable, with a free energy minimum of −20 kcal/mol [14].

### 2.2. Adsorption Isotherms

In order to relate the PMF data above to more intuitive physical quantities, we have devised a simple statistical-mechanical model to calculate membrane adsorption and insertion isotherms of PG-1. We are interested in computing the equilibrium concentrations of the various membrane-associated states of protegrin: The bulk concentrations of monomers (*c*_M,B_) and dimers (*c*_D,B_); the surface density of adsorbed monomers (*ρ*_M,S_) and dimers (*ρ*_D,S_); and the surface densities of membrane-inserted monomers (*ρ*_M,I_) and dimers (*ρ*_D,I_). All of these quantities are computed as a function of the total peptide concentration, *c*_o_. The model consists of expressing all the relevant processes as reversible chemical reactions and equating the chemical potentials of the products and reactants. The chemical potentials are in turn calculated based on standard statistical mechanical methods [15–17], which naturally leads to the single-molecule PMFs. The derivation is straightforward, but has been omitted here for the sake of brevity. The resulting system of equations relating the quantities of interest is shown below.

(1) $${\text{c}}_{\text{D},\text{B}}=\frac{1}{16\pi^{2}}{\text{c}}_{\text{M},\text{B}}^{2}\int_{\text{dimer state}}{\text{e}}^{-\beta {\text{W}}_{\text{dim},\text{B}}(\text{r})}\;{\text{r}}^{2}\;\text{dr}$$

(2) $$\rho_{\text{M},\text{S}}^{L}=\left(1+\sum_{k}\rho_{k}^{L}\bar{{\text{A}}_{k}}\right)\frac{{\text{c}}_{\text{M},\text{B}}}{4\pi }{\text{e}}^{-\beta \mu_{\text{M},\text{S}}^{R}}\int_{\text{adsorbed state}}{\text{e}}^{-\beta {\text{W}}_{\text{M},\text{ads}}(\text{z})}\;\text{dz}$$

(3) $$\rho_{\text{D},\text{S}}^{L}=\left(1+\sum_{k}\rho_{k}^{L}\bar{{\text{A}}_{k}}\right)\frac{{\text{c}}_{\text{M},\text{B}}}{4\pi }{\text{e}}^{-\beta \mu_{\text{D},\text{S}}^{R}}\int_{\text{adsorbed state}}{\text{e}}^{-\beta {\text{W}}_{\text{D},\text{ads}}(\text{z})}\;\text{dz}$$

(4) $$\rho_{\text{M},\text{S}}^{L}=\rho_{\text{M},\text{I}}^{L}\frac{\int_{\text{ads}.\;\text{state}}{\text{e}}^{-\beta {\text{W}}_{\text{M},\text{ins}}(\text{z})}\;\text{dz}}{\int_{\text{ins}.\;\text{state}}{\text{e}}^{-\beta {\text{W}}_{\text{M},\text{ins}}(\text{z})}\;\text{dz}}{\text{e}}^{-\beta \;(\mu_{\text{M},\text{S}}^{R}-\mu_{\text{M},\text{I}}^{R})}$$

(5) $$\rho_{\text{D},\text{I}}^{L}=\frac{{\rho_{\text{M},\text{I}}^{L}}^{2}}{4\pi (1+\Sigma_{k}\rho_{k}^{L}\bar{{\text{A}}_{k}})}{\text{e}}^{-\beta \;(\mu_{\text{M},\text{D}}^{R}-2\;\mu_{\text{M},\text{I}}^{R})}\int_{\text{dimer state}}{\text{e}}^{-\beta {\text{W}}_{\text{dim},\text{I}}(\text{r})}\;\text{r dr}$$

(6) $$c_{\text{M},\text{B}}+2c_{\text{M},\text{B}}=c_{\text{o}}$$

The superscript *L* for different surface densities denotes that surface densities are defined based on the total area covered by lipids (as opposed to the expanded area due to inserted species). *Ā_k_* denotes the specific lateral area of an inserted species *k* in units of Å^2^, while ρ*_k_^L^* denotes the surface density in units of Å^−2^. The summations over the index *k* in (2), (3) and (5) are performed only over the inserted species (*i.e.*, inserted monomers and dimers). The various PMFs are denoted as follows: *W*_dim,B_, the PMF of dimerization in the bulk phase; *W*_M,ads_ and *W*_D,ads_ the PMFs of monomer and dimer adsorption, respectively; *W*_M,ins_, the PMF of monomer insertion; *W*_dim,I_, the PMF of dimerization in the membrane interior. The limits of integration indicate which regions of the PMF should be included; while this is somewhat arbitrary, the precise bounds of any particular state are not important, since the largest contribution to the integral arises from the region near the minimum. Finally, *μ_i_^R^* represents the excess chemical potential due to area exclusion/crowding effects; we have used the model of Talbot [16] for multicomponent mixtures in a two-dimensional domain, which is based on the scaled particle theory approach of Reiss *et al*. [18]. The excess chemical potential *μ_i_^R^* of species *i* is given by (7) as a function of the surface densities *ρ_j_*, circumferences *L_j_* and lateral areas *A_j_* of all membrane-associated species. *A_j_* and *L_j_* are based on best-fit ellipses of the planar projections of the relevant states, extracted from MD simulations.

(7) $$\beta \mu_{\text{i}}^{\text{R}}=-\text{ln}\;\left(1-\sum_{\text{j}}\rho_{\text{j}}{\text{A}}_{\text{j}}\right)+\frac{{\text{A}}_{\text{i}}\;\Sigma_{\text{j}}\;\rho_{\text{j}}+{\text{L}}_{\text{i}}\;\Sigma_{\text{j}}\;\rho_{\text{j}}{\text{L}}_{\text{j}}/2\pi }{1-\Sigma_{\text{j}}\;\rho_{\text{j}}{\text{A}}_{\text{j}}}+\frac{{\text{A}}_{\text{i}}\;{\left(\Sigma_{\text{j}}\;\rho_{\text{j}}{\text{A}}_{\text{j}}\right)}^{2}}{4\pi \;{\left(1-\Sigma_{\text{j}}\;\rho_{\text{j}}{\text{A}}_{\text{j}}\right)}^{2}}$$

**Figure 3:**
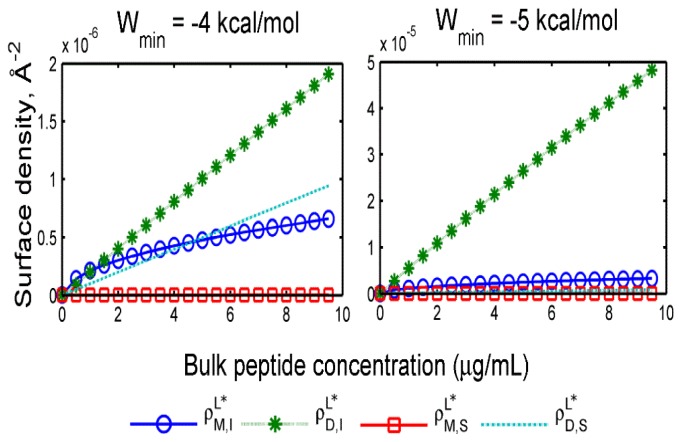
Membrane association isotherms for insertion free energies of −4 kcal/mol (**left**) and −5 kcal/mol (**right**).

As already mentioned, we have only recently computed the PMF of insertion. We therefore also present results where the insertion free energy is left as a free parameter (*W*_min_), and a parabolic well shape is assumed for the well depth. Figure 3 below shows adsorption and insertion isotherms for two values of the insertion free energy minimum.

**Figure 4:**
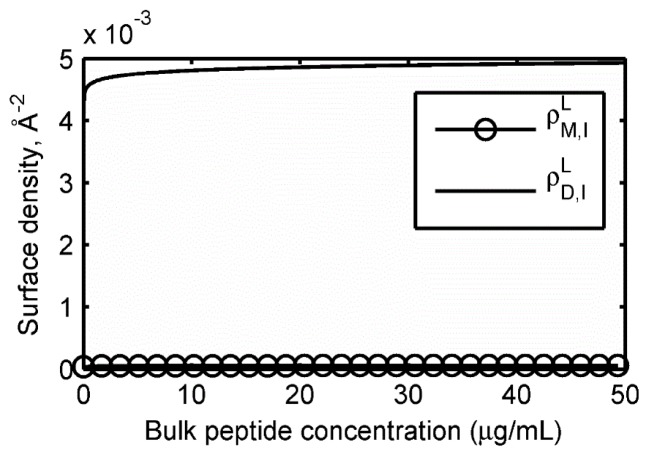
Membrane association isotherm for monomers and dimers based on the free energy of insertion computed from MD simulations (corresponding to *W*_min_ = −20 kcal/mol).

At free energies of −4 kcal/mol, inserted dimers, surface-bound dimers and inserted monomers are all present in comparable amounts. With only a slight decrease in the insertion free energy to −5 kcal/mol, inserted dimers become the dominant state. In Figure 4, we show the results of the same calculations based on the insertion PMF recently computed with MD simulations, corresponding to *W_min_* = −20 kcal/mol.

Not surprisingly, inserted dimers completely dominate the isotherms, and the high surface density leads to almost complete saturation due to crowding effects. Although rudimentary in their treatment, these models offer a novel perspective on the key membrane association steps in the action of protegrin, from molecular-level thermodynamics to macroscopically relevant quantities such as adsorption isotherms.

### 2.3. Protegrin Pores and Ion Transport Models

In this section, we turn our attention to protegrin transmembrane pores, the structural units responsible for the antimicrobial properties of protegrins, as well as many other AMPs [3,5,19,20].

#### 2.3.1. Molecular Simulations of Protegrin Pores

**Figure 5:**
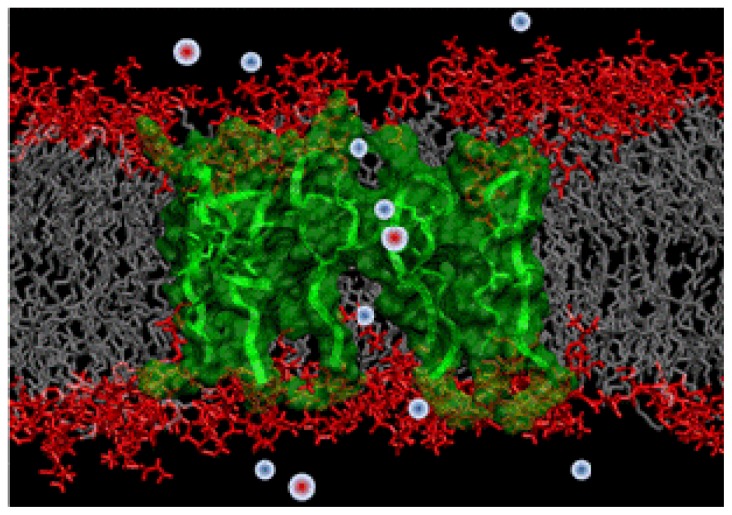
Snapshot from molecular dynamics simulations of an antimicrobial peptide pore (peptides are shown in green) inside a cell membrane (lipid headgroups are shown in red and lipid acyl chains in gray). When a pore is formed, vital sodium and chloride ions (blue and red spheres) move in and out of cells too fast for bacteria to respond, leading to cell death. Computer simulations can provide structural and dynamic information of the relevant transport phenomena impossible to achieve with current experimental methods.

Our first foray into the investigation of these structures consisted of fully atomistic MD simulations of an octameric protegrin pore embedded in a lipid bilayer [9]. The starting structure for these simulations was based on NMR experiments reported by Mani *et al*. [21], which suggest that protegrin pores are most likely composed of four parallel dimers. We first started with simulating monomeric and dimeric structures in various membrane mimic environments [22–40]. Ultimately, we simulated the pore structure for more than 150 ns, and found it to be indeed stable, with good agreement between NMR-derived inter-peptide distances and corresponding simulation data (Figure 5). Our simulation showed that the pore opening is large enough to readily conduct anions, but rarely allows cations to pass through due to the large positive charges of the peptides.

#### 2.3.2. Continuum Electrodiffusion Models

Based on the results of our MD simulations, we conjectured that the unrestricted transport of ions facilitated by protegrin pores would disrupt the transmembrane potential of bacteria, resulting in cell death. To investigate this point further, we first devised and implemented a modeling strategy to compute the single-channel conductance properties of protegrin pores. Our goal was to provide a quantitative connection between earlier patch clamp experiments by Sokolov *et al*. [6] and atomistic-level knowledge of pore structures from MD simulations [9] and NMR experiments [21].

**Figure 6:**
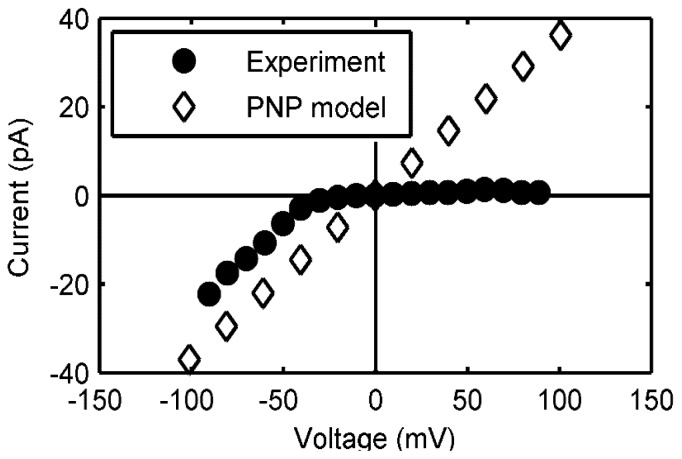
Current-voltage relationship of a protegrin pore.

In order to circumvent the computational difficulties associated with nonequilibrium MD simulations, we employed a continuum transport theory known as the Poisson-Nernst-Planck (PNP) equations [22]. We extract snapshots from the equilibrium MD simulation [9], which retain atomistic detail for the peptide and lipids, but treat the ions as continuum concentrations around the rigid pore structures. In steady-state conduction, the model consists of the following Equations:

(8) $$\nabla \cdot \left({\text{D}}_{\text{i}}\nabla {\text{c}}_{\text{i}}+\frac{{\text{D}}_{\text{i}}{\text{q}}_{\text{i}}\text{e}}{{\text{k}}_{\text{B}}\text{T}}{\text{c}}_{\text{i}}\nabla \phi \right)=0,\;\;\;\text{i}={\text{K}}^{+},{\text{Cl}}^{-}$$

(9) $$\nabla \cdot (ɛ\nabla \phi )=-\rho_{\text{f}}-\sum_{\text{i}=1}^{\text{N}}{\text{q}}_{\text{i}}{\text{c}}_{\text{i}}$$

where *c_i_*, *D_i_* and *q_i_* are the concentration, diffusivity and valence of each ionic species, ϕ is the electrostatic potential, *e*, *k_B_* and *T* are the elementary charge, Boltzmann’s constant and temperature, respectively, ɛ is the space-dependent dielectric constant, and ρ*_f_* is the fixed charge density. The electrostatic potential is obtained by solving the Poisson equation (9) consistently with (8), and the net current is determined based on the resulting ion fluxes. The computed current-voltage (I-V) relationship is shown in Figure 6 below, along with experimental data from [6].

The flat portion of the experimental I-V curve suggests a voltage-dependent gating, wherein the protegrin pore collapses at positive voltages. Since our model assumes the pore to be a rigid boundary for the PNP equations, we cannot capture this. However, as long as the pore is open, the match in the slopes of the two curves, which is equivalent to conductance, is excellent. This work further corroborates the structure of protegrin pores, and offers a direct quantitative connection between ion transport properties and atomistic-level structural features of the pore.

### 2.4. Ion Transport from an Entire Bacterial Cell

**Figure 7:**
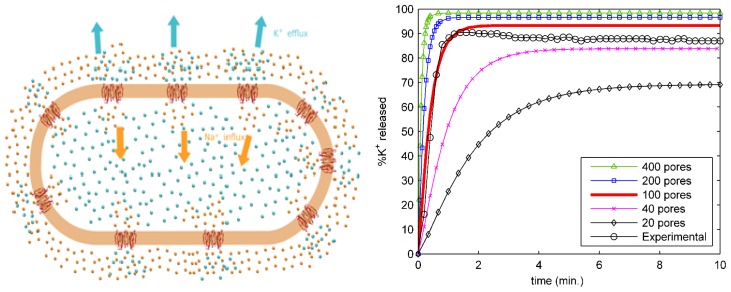
**Left** panel: schematic of a whole bacterial cell model. Protegrin pores (in red on the orange cell membrane) induce rapid transport of potassium (cyan spheres) and sodium (orange spheres) ions outside and inside the cell, respectively. From [11] with permission. **Right** panel: Potassium release curves for multiple values of the number of pores, along with experimental data. From [10] with permission.

Our success in modeling the single-channel conductance of protegrin pores led us to ask what the effects of such pores would be on an entire cell. Using the single-pore conductance obtained from our PNP model, we have constructed a larger scale model that yields the time-dependent ion concentration in bacterial cells [10]. This model treats the bacterial interior and surrounding bath as well-mixed volumes with respect to ion diffusion, which allows for a simple, space-independent description of transport. The total flux of each ionic species is a function of the single-pore permeability values, which are obtained from the 3D-PNP calculations discussed above, as well as the number of pores, which is treated as a variable parameter. The model is described in detail in [10]. By adjusting only the number of pores in our model, we were able to match experimentally measured potassium leakage data from live exponential-phase *E. coli*, and thus provide the first estimate of the number of pores required to kill an *E. coli* cell—approximately one hundred. Potassium release curves are shown in Figure 7 below for different numbers of pores.

**Figure 8:**
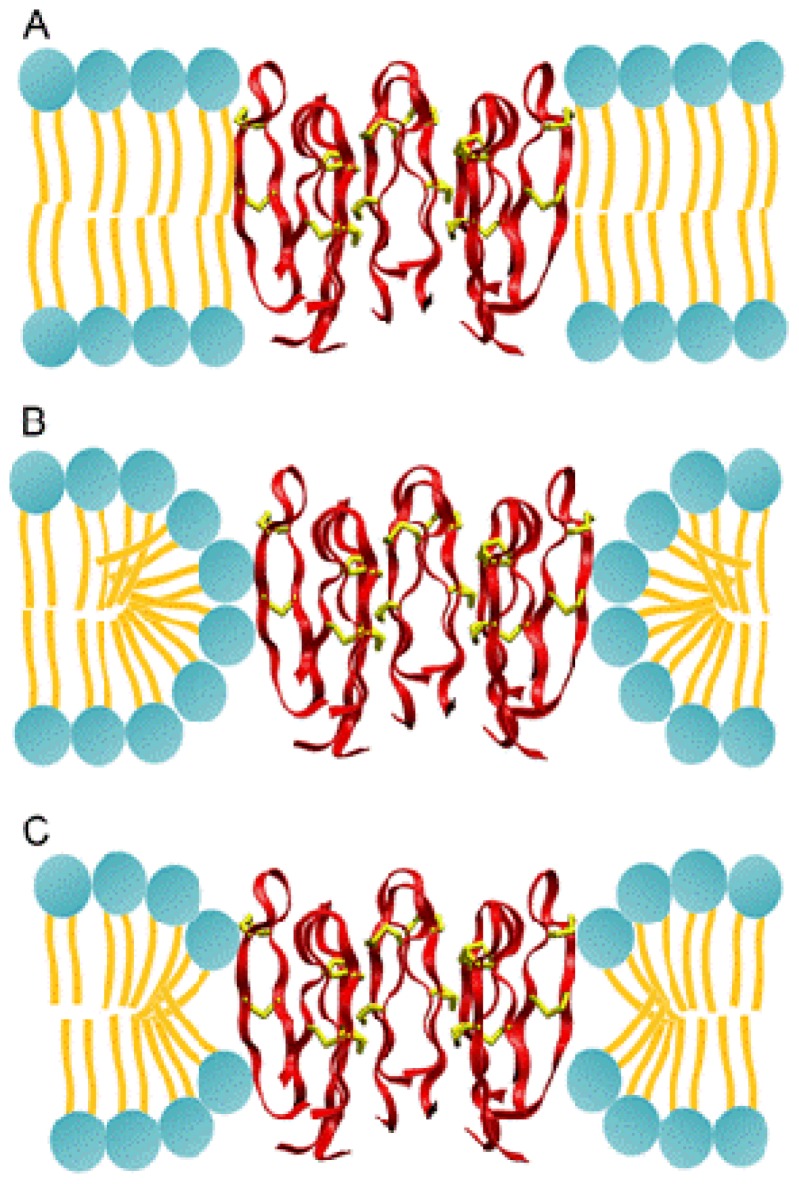
The different models of lipid topologies surrounding the PG-1 transmembrane pore. (**A**) The barrel-stave pore, where lipids retain their alignment with the bilayer normal; (**B**) The toroidal pore, where lipids tilt fully towards the pore to create a continuous leaflet that completely lines the outside of the pore; (**C**) The semi-toroidal pore, where lipids tilt partially towards the peptides, but do not form a continuous leaflet. From [11] with permission.

One important remaining question is related to the structure of lipids around the protegrin pore. Determining the structure of lipid bilayers is an important determinant of activity and specificity of protegrins. These antimicrobial peptides are known to be active against Gram negative bacteria but not so active against Gram positive bacteria. A hypothesis is that the lipid membranes of various bacteria have different compositions of lipid molecules that result in different energies for pore formation. In Figure 8, the three prevalent structures are shown. Although it is currently not clear how the lipid composition impacts the pore formation free energies, we believe that molecular simulations may provide useful insight into the molecular interactions than underlie antimicrobial peptide activity and specificity.

## 3. Conclusions

The current knowledge of the mechanism of action of protegrin, while more expansive than for almost any other AMP, still lacks a quantitative, physics-based perspective. In particular, while biophysical studies have isolated and elucidated several important phenomena (e.g., membrane association, dimer formation, pore formation, ion conduction), few attempts have been made to provide a unified analysis that connects these different important aspects of the protegrin mechanism of action. The multiscale analysis presented herein makes a number of significant contributions to the current understanding of this mechanism of action. We hope that this work stands as proof of the usefulness of quantitative, physics-based approaches in the investigation of complex biological systems, particularly in their ability to connect distinct but coupled phenomena across disparate time and length scales.
